# Effects of glycosylated hemoglobin levels on neutrophilic phagocytic functions

**DOI:** 10.5897/JDE2017.0110

**Published:** 2017-07-31

**Authors:** Mary Michelle Shodja, Raymond Knutsen, Jeffrey Cao, Keiji Oda, Lawrence E. Beeson, Gary E. Fraser, Synnove Knutsen

**Affiliations:** 1 Center for Nutrition, Healthy Lifestyle and Disease Prevention Faculty, Loma Linda, California, United State; 2 School of Medicine Loma Linda University, California, United State

**Keywords:** A_1_c, hemoglobin A_1_c, phagocytosis, neutrophils, phagocytic index, diabetes, infection

## Abstract

It is well established that diabetic patients with poor glycemic control have increased susceptibility to infections, but glucose levels have not been directly associated with this increase. The assessment of the effects of glycosylated hemoglobin (A_1_ c) on the body’s ability to fight infections may be useful directly in establishing a link between elevated blood sugar and the risk of infections. A total of 127 subjects in Heart Pilot Study (HPS), sub-study of the Adventist Health Study 2 (AHS-2) completed a lifestyle, medical and food frequency questionnaire (FFQ) at baseline between 2013 and 2014. The A_1_ c and phagocytic index (PI) were measured in the same blood sample and their associations were assessed using linear regression. Mean blood glucose (MBG) was estimated based on A_1_ c levels using a standard formula. Three levels of MBG were used to compare prediabetic and diabetic ranges to the normal range. The PI is the average number of bacteria in the cytoplasm of 50 neutrophils, manually counted under a light microscope after the whole blood was briefly exposed to a standard dose of bacteria and stained. In multivariable analysis, we found that MBG in the prediabetic (117 to137 mg/dL) and diabetic (>137 mg/dL) ranges were associated with 12.9% (β= −0.129, 95% Cl: −0.30, 0.05) and 20.4% decrease in PI (β= −0.204, 95% Cl: −0.592, 0.184) compared to that, observed among those with normal MBG (*p* for trend=0.119). Elevated MBG levels contribute a decrease in the PI among those in the prediabetic and diabetic range compared to the normal range. Although our findings were not quite statistically significant due to low power which are clinically relevant in line with observations of an increased infections among diabetics. Further research on larger populations is needed.

## INTRODUCTION

Infectious disease remains a health burden in any population but in general, are more frequent and/serious in patients with diabetes mellitus (Casqueiro et al., 2012). Serious infections that are unique to or more complicated in patients with diabetes include soft tissue infections, urinary tract infections and other miscellaneous infections such as rhinocerebral mucormycosis and emphysematous cholecystitis (Paauw, 2000).

It is well established that processed sugars indirectly increase the risk of diabetes; they play a major role in the development of obesity (Johnson et al., 2007). More directly, a recent study (Basu et al., 2013) found that for every 150 kcal/day/person increase in sugar intake (equivalent to a 12 oz soda daily), diabetes prevalence increases by 1.1%. It is also widely known that diabetic patients with poor glycemic control are susceptible to infections (Janifer et al., 2009; Rueda et al., 2010; Saengmuang et al., 2014) and it seems probable that elevated levels of glucose in the blood of these patients somehow contributes to the decrease in resistance to infection.

One of the first studies that explored the possibility which decreased phagocytic activity may be related to the increased risk of infections. Richardson (1942) reported the ability of leukocytes, mainly the neutrophils in the blood, to phagocytize bacteria. Richardson’s study found that phagocytosis is not affected in the controlled diabetic patient (even with fluctuations in blood sugar that are commonly found in diabetic patients), but was significantly decreased in diabetic patients with complications of acidosis and marked malnutrition.

Similarly, Van Oss (1971) reported that high serum glucose levels (200, 400 and 800 mg/100 ml) significantly decreases the neutrophilic phagocytosis of *Staphylococcus epidermidis, Staphylococcus aureus* and *Escherichia coli.* He also noted that there is a decrease in adhesiveness of the neutrophils to solid surfaces with increasing levels of glucose of 200, 400, and 800 mg/100ml.

Speert et al. (1984) reportded that phagocytosis of *Pseudomonas aeruginosa* was significantly inhibited when mannan (a plant-derived, complex polysaccharide mainly of polymers of the sugar mannose), D-fructose, α-methyl-D-mannoside, D-mannose, or D-glucose was present in the phagocytosis mixture (Moreira and Filho, 2008). In addition, the researchers also found that the inhibitory effect was reversible (Speed et al., 1984).

White blood cells (WBCs) include 5 different types of cells like, neutrophils, lymphocytes, monocytes, eosinophils and basophils. Neutrophils (neutral staining cells) or polymorphonuclear (PMN) leukocytes (“poly” because the nucleus is segmented), together with monocytes function mainly as the primary innate defenses against intrusion of infectious agents and other foreign materials into the host environment (Greer, 2013). Neutrophils are a part of the body’s innate immunity against infections because of their ability to phagocytize and destroy bacteria. Phagocytosis is an energy requiring mechanism that is inhibited by inadequate supply of glucose, but Sanchez et al. (1973) reported that an excess of glucose also decreases phagocytic activity. This observation is supported by recent studies (de Souza Ferreira et al., 2012; Lecube et al., 2011).

According to Van Oss (1971), many factors affect phagocytosis and the phagocytic capacity of neutrophils of a normal subjects may vary from day to day. Such factors include lower temperatures (Mondal and Rai, 2001), increased hydrogen-ion concentration (Marion et al., 2004), increased cytoplasmic viscosity (Marion et al., 2004), and the presence of certain substances such as iodine (Stolc, 1972) or lactic acid (Ene et al., 2013). *In-vitro* addition of histamine (Laszlo et al., 2001) or calcium ions (Diler et al., 2014), exposure to interferon (Huang et al., 1971) or stimulation of toll-like receptors (TLRs) (Ribes et al., 2010) appear to increase the phagocytosis.

Research that has examined the association of glycosylated hemoglobin (A_1_c) and phagocytic functions of neutrophils is limited to only one study by Saito et al. (2013) who repoded a negative correlation between levels of A_1_ c and stimulated reactive oxygen production (ROS) (p = 0.072) and phagocytic activity (PA) (p = 0.059). With the growing evidence that elevated sugar in the blood or hyperglycemia decreases neutrophilic functions and thus resistance to infections, we sought to evaluate the effects of A_1_ c levels on the neutrophilic phagocytic function. In order to shed light on the role of blood sugar on the body’s susceptibility to infections, we studied 127 non-diabetic subjects who were participants in the Heart Pilot Study (HPS), a sub study of the larger Adventist Study-2 cohort (Butler et al., 2008).

## MATERIAL AND METHODS

### Study population

The HPS is a small cardiac ultrasound study conducted within the larger prospective Adventist Health Study 2 (AHS-2) (Butler et al., 2008), which enrolled approximately 96,000 subjects from all 50 US states and 5 provinces of Canada from 2002 through 2007. Participants completed a 50-page enrollment questionnaire on lifestyle characteristics and other demographic variables (Retrieved. 2017January28. http://publichealth.llu.edu/sites/publichealth.llu.edu/files/docs/sph-adventist-health-study-2.pdf) as well as a validated food frequency questionnaire (FFQ)(Jaceldo-Siegl et al., 2010) and medical history. The process of subject recruitment is described in detail elsewhere (Butler et al., 2008).

The HPS that began in the fall of 2013 and ended in the spring of 2014 is a small pilot sub-study of the AHS-2 that aimed to assess the role of diet and lifestyle on risk of sub-clinical heart diseases. Subjects were recruited from the AHS-2 master list of subjects, which was narrowed down into a shorter list of those living in California between the Loma Linda and Los Angeles area.

Subjects were randomly selected and contacted by telephone, ensuring that there were equal proportions of Black and White subjects who were aged 50 years and older and 50% being females. Subjects were invited to a clinic where they, in addition to the clinic components, completed a new diet questionnaire (“The Heart Pilot Questionnaire, 2013”), which was identical to the AHS-2 baseline questionnaire with regards to a FFQ and physical activity, but also included information on smoking, physical activity and medical history.

**Figure 1.**
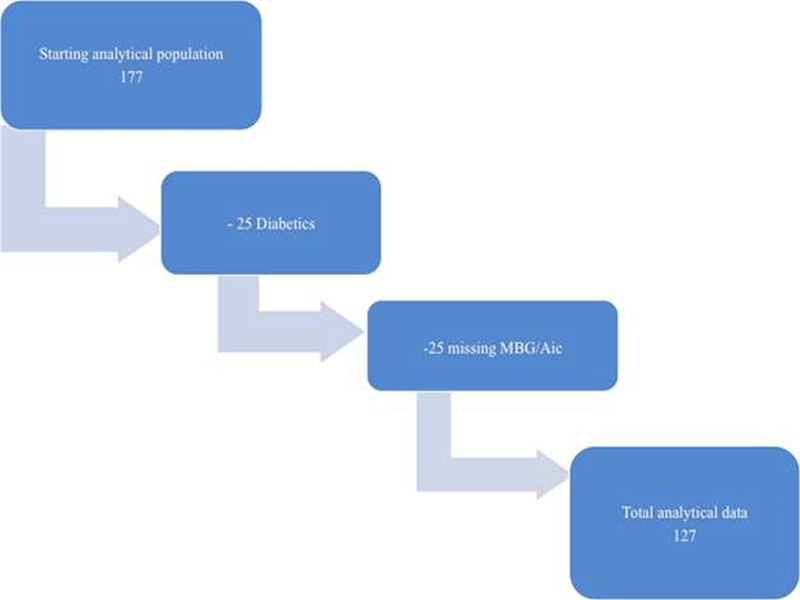
Chart of the final analytical models.

Among the 200 in the HPS study, blood samples were available from a total of 177 subjects. After exclusion of known diabetics (n=25) and missing values for mean blood glucose (MBG)/or HbA_1_C (n=25), a total of 127 individuals remained for analysis (Figure 1).

### Exposure estimates

Glucose attaches to the hemoglobin portion of the red blood cells through a process called glycation or glycosylation (Tietz, 1976). Hemoglobin with glucose attached to it is referred to as hemoglobin A_1_c (A_1_c), glycosylated hemoglobin or glycated hemoglobin (Tietz, 1976). Since the average life of a red blood cell is approximately 100 to 120 days (Hillman and Hillman, 2011), the measurement of A_1_c will give a MBG level for the past 4 months which is viewed as a more stable way to monitor blood sugar levels compared to fasting blood sugar (FBS) which tends to fluctuate from day to day.

Blood was obtained though venipuncture in each HPS participant and the same blood was used for assessment of serum glucose, A_1_ c and phagocytic index (PI). The A_1_ c test was conducted in the Medical Sciences Laboratory, a California licensed clinical laboratory located in Anaheim, California, using the Bio-Rad D-10™ high performance liquid chromatography (HPLC). The A_1_c values were converted into MBG expressed in mg/dL by multiplying the A_1_c value by 28.7 and subtracting 46.7 (Retrieved.2016June9. http://www.globalrph.com/hba1c.htm). The normal MBG range is less than 117 mg/dL, the prediabetic range is 117 to 137 mg/dL and the diabetic range is greater than 137 mg/dL (Retrieved.2017January7. http://www.mayoclinic.org/tests-procedures/a1c-test/details/results/rsc-20167939).

### Phagocytic index

Neutrophilic phagocytosis was chosen for the PI over monocytic phagocytosis because neutrophils comprise approximately 40 to 70% of all WBCs in the blood compared to 2 to 10% for monocytes (Greer, 2013). Neutrophils eliminate foreign invaders through the process of phagocytosis (Lee et al., 1993) wherein bacteria undergo receptor-mediated ingestion or endocytosis and are digested in the resulting phagosome (Lee et al., 1993) which can be measured by PI (Sanchez et al., 1973). The PI was assessed at the Medical Sciences Laboratory using the same blood sample used to assess the A_1_c test employing a modified manual procedure described by Sanchez et al. (1973). In addition, PI result validation was performed at Loma Linda University Medical Center Laboratory on 23% of the samples.

The PI validation consisted of randomly selecting 29 slides; recounting the bacteria in the cytoplasm of the first 50 neutrophils by 2 different Clinical Laboratory Scientists (CLSs) specialized in the areas of hematology and microbiology at Loma Linda University Medical Center, Loma Linda, California.

To perform the PI test, 900 μl of whole blood collected in ethylenediaminetetraacetic acid (EDTA) is mixed with 100 μl of a 0.5 McFarland suspension (yielding approximately 1.5 χ 10^8^ bacterial cells) (McFarland, 1907) of Staphylococcus epidermidis, American Type Culture Collection (ATCC 12228) (Clinical and Laboratory Standards Institute, 2016). The mixture is then placed in a mechanical rotator at 100 rotations per minute (RPM) for 30 min at 37°C. A blood smear is then prepared from the mixture and stained with the Wright’s™ stain manufactured by Fisher Scientific, Waltham, MA. The PI is the mean number of bacteria in the first 50 neutrophils under a light microscope with 2500x magnification. The PI has no established normal range but the higher the index, the higher the phagocytic activity and the more indicative of a well-functioning immune system (Muniz-Junqueira et al., 2003).

In addition, a complete blood count (CBC) was performed on all samples using the EDTA preserved whole blood to assess each participants’ WBC levels. Testing each subject’s WBC count and including those subjects with normal range of 3.8 to 11.5 (mm^3^), (Greer, 2013) will ensure that the low PI result is not due to low WBC count or that a high PI result is not due to excess WBC in the blood. All 127 subjects in the study have WBC results within the normal range.

### Covariates

Potential covariates were identified priori and based on previous studies (Falagas and Kompoti, 2006; Falagas et al., 2007; Friman and Wesslen, 2000; Gamble et al., 2006; Lampe, 2003; Schraufnagel et al., 2013), known associations, and availability in the HPS database. The following variables were identified and are listed in Table 1: age (4 levels), gender (dichotomous), race (Blacks/non-Blacks), physical activity [(exercisers) those who exercise more than 1 h a week / (non-exercisers) those who exercise less than 1 h a week)], body mass index (BMI) (3 levels), alcohol consumption (ever/never) and dietary pattern (vegetarian/non-vegetarian).

The vegetarian group consists of vegans who consume meat (red meat, poultry and fish combined) < 1/month and dairy products < 1/month, the lacto-ovo vegetarians are those who eat any meats or fish < 1/month, but consume eggs and dairy products, the semivegetarians are those who consume meat and fish for up to four servings a month, and the pesco-vegetarians are those who eat meat < 1/month, but eat fish once a month or more. The nonvegetarians eat meat once a week or more.

### Statistical analysis

The Statistical Analysis Software (SAS) version 9.4 (Statistical Analysis Software, North Carolina, USA), was used for all analyses. The R-studio version 3.2 (Boston, MA) was used to perform a guided multiple imputation for missing BMI and exercise data as well as for PI validation. To validate the PI test, a widely used reliability index in the interrater reliability analysis, the Intraclass correlation coefficient one-way random effects model [ICC (1,1)] (Koo and Li, 2016) was performed on the three PI measurements (the measurement used for the analysis and the two repeated measurements).

Chi-square was used to assess descriptive characteristics of the population according to the MBG levels and a one-way ANOVA was used to calculate the Pi’s mean and standard deviation (SD) for each of the 3 levels of MBG. The continuous outcome PI was log transformed to minimize the slightly right skewed data. The total analytic population of 127 allowed the inclusion of all 7 covariates.

To assess the association between the three MBG levels and PI, linear regression was performed using PROC GLM to assess the percent difference in PI (treated as a continuous variable) among those in the prediabetic and diabetic MBG range compared to the normal range. First, we did an age-adjusted model and then a full model with all seven candidate covariates. Finally, two sensitivity analyses were performed, first using MBG as a continuous exposure and secondly where the 25 prevalent diabetics were included in the “diabetic range group” of the analytic population.

## RESULTS

Among the 127 subjects with MBG estimated from the A_1_c results, 71 (56.0%) have normal MBG, 50 (39.3%) have prediabetic MBG and only 6 subjects (4.7%) have diabetic MBG values. No significant differences in demographic characteristics were found between the three MBG level groups (normal, pre-diabetic and diabetic) although, the PI tended to be higher among those in the normal range. Table 1 presents the characteristics of the participants at baseline according to the 3-level (normal, prediabetic and diabetic) MBG range. The PI validation of 29 out of 127 slides (23%) showed an ICC (1,1) = 0.844, 95% Cl (0.736 to 0.917) indicative of good reliability.

The association between MBG levels and PI showed a negative trend (Table 2) in the age-adjusted model. Compared to subjects in the normal MBG levels, there was a non-significant monotonic trend (p=0.109) with those in the pre-diabetic and diabetic range of MBG having 13.0 and 16.0% lower PI, respectively, and an R^2^ of 0.053 (Table 2). In the full multivariable model, the findings were slightly strengthened with PIs of 12.9 and 20.4% lower, respectively, for pre-diabetic and diabetic MBG. The R^2^ also slightly improved to 0.131 while the p for trend was virtually unchanged (Table 2).

The results of the sensitivity analysis using MBG as a continuous exposure showed a lower R-square compared to the categorical MBG therefore, the latter was selected for analysis. The second sensitivity analysis including those with diagnosed diabetes actually resulted in a much higher PI estimate in the diabetic group compared to when diabetics were excluded from this group. Thus, there was no longer clear trend across the MBG groups. None of the covariates were independent predictors of PI when included in the multivariable model (Table 3).

## DISCUSSION

To the best of our knowledge, this paper is the first to look specifically at the relationship between MBG levels and phagocytic functions as measured by the manual PI test among non-diabetics. However, some have looked at similar exposure measures. Sanchez et al. (1973) performed a 5 h carbohydrate tolerance (glucose, fructose, sucrose, honey and starch) test where subjects were placed in a group that ingested 100 g of a specific carbohydrate. A baseline and 5 hourly blood draws were performed on each subject and a manual PI test was done on each specimen. Sanchez et al. (1973) reported a decrease in PI after ingestion of glucose, fructose, sucrose and honey with the greatest effect observed around 1 to 2 h post carbohydrate ingestion. The PI was normalized around the 5^th^ hour.

More recently in 2013, Saito et al. (2013) assessed the association of HbA1c and neutrophil function in a Japanese general population. In this study, exposure of blood to hydroethidine stimulates the neutrophil production of reactive oxygen species (ROS), which is then measured in a flow cytometry. ROS is produced during neutrophilic phagocytosis and an increase in ROS production after stimulation indicates properly functioning neutrophils. Similar to our findings, Saito reported an inverse, non-statistically significant finding (p=0.092) between HbA1c and neutrophilic function, observed that there was an 8.1% decrease in phagocytic activity (PA) among females compared to males. A similar conclusion that hyperglycemia decreases phagocytosis was reported by Perner et al. (2003). In addition, Wilson and Reeves (1986) reported that those with type 2 diabetes have decreased phagocytic functions.

Our research attempts to establish whether elevated MBG possibly affects the immune system through its effects on phagocytic function as measured by PI. Although there is a growing number of papers indicating that chronic hyperglycemia negatively affect phagocytic functions, further investigation is needed. One of the long-term effects of elevated MBG/A_1_c as seen among subjects with uncontrolled diabetes increased susceptibility to infections due to reduced response of T-cells, neutrophil function and disorders of humoral immunity (Casqueiro et al., 2012). A normal A_1_ c is below 5.7%, an A_1_ c level of 5.7 to 6.4% is considered prediabetic levels and greater than 6.4% is considered diabetic levels.

The results of the sensitivity study that includes subjects with previously diagnosed diabetes showed attenuation towards the null in the PI estimate compared to when diabetics were excluded. One explanation why the diabetics in this study did not have a decreased PI may be due to the fact that the subjects in this study had well-controlled diabetes with a mean A_1_ c of 7.1%. The recommendation for individuals with diabetes is maintaining an A_|_ c level of <7.0% in conjunction with a healthy lifestyle and diet (Sherwani et al., 2016). Thus in our study, diabetic group would tend to attenuate the results since a large proportion of them (32%) were not in the diabetic range based on their A_1_ c.

Limitations of this study include low power due to the small number of subjects. A larger study size may have provided more definitive answers to our research question. Although the PI validation showed good reliability between slide readers, the manual procedure of counting bacteria in the cytoplasm of the neutrophil is not only time consuming, but may be more prone to error if performed by inexperienced individuals. Analysis using flow cytometry will give a more accurate and reliable result, especially when there is a high volume of specimens. The participants of this study were over 50 years old and may have had a diminished immune function just by being elderly therefore inclusion of other age groups is recommended.

The strengths of the study include a relatively healthy population of only 20% obese (BMI (>30 kg/m^2^) with the majority being non-alcohol consumers and over 80% are never smokers with the remaining being past smokers. Alcohol consumption and smoking are major confounders in most statistical analysis on health and this population is therefore unique in being virtually free from these confounders. Also, instead of just a random measure of FBS, we were able to study the effects of chronic hyperglycemia through A1 c and MBG. Although there is a short-term effect of high glucose levels on PI levels, it seems to disappear after a few hours (Sanchez et al., 1973). However, the effect of chronic elevated blood glucose levels is probably not transient. Thus, chronic hyperglycemia, as measured by A_1_ c in this study, seems to have a significant and clinically relevant effect on PI levels and thus on resistance against infections. Based on our findings, A_1_ c is probably a better measure of the relationship between blood glucose levels and PI than a random blood glucose measurement.

### Conclusion

This is one of very few studies that have studied the relationship of MBG through the measurement of HbA_1_c and phagocytic function as measured by the PI. Our findings support and possibly give a mechanism for the reports of increased infections among diabetics. The findings of a clinically relevant decrease in PI with increasing MBG levels is directly relevant in patient care and support and possibly gives a mechanism for the increased infection rates among diabetics. Thus our findings are generally supportive and provide evidence that, chronic elevated sugar in the blood affects neutrophilic phagocytosis, which may affect our ability to fight infections.

Recent studies have also linked glycemic status as well as immune function to stroke outcome (Lattanzi, et al., 2016) including neurological deterioration after acute cerebral hemorrhage (Lattanzi et al., 2017). Thus our findings may have relevance for clinical outcomes beyond risk of infections. These findings in line with the pattern of decreasing PI, with an increasing MBG levels should be further explored with a larger sample size which includes a variety of age groups.

## Figures and Tables

**Table 1.** Population Characteristics of the 3-level outcome MBG range.

Study population characteristics	Mean Blood Glucose (MBG)			p-value
	Normal <117 mg/dL (n=71)	Pre diabetic range (117–137 g/dL) (n =50)	Diabetic range (>137 mg/dL) (n=6)	
PI *	8.7 (4.6)	7.8 (3.3)	7.5 (4.2)	0.262
Age	(%)	(%)	(%)	0.989
50–69	38.0	42.0	33.3	
70–79	31.0	30.0	33.3	
80–89	25.4	22.0	33.4	
90+	5.6	6.0	0.0	
Gender				0.193
Female	54.1	50.0	16.7	
Male	45.1	50.0	83.3	
Race				0.152
Blacks	33.8	48.0	16.7	
Whites	66.2	52.0	83.3	
Exercise				0.389
≤1 hr/week	38.0	40.0	66.7	
>1 hr/week	62.0	60.0	33.3	
BMI				0.929
<25	43.7	40.0	50.0	
25–30	36.6	44.0	33.3	
>30	19.7	16.0	16.7	
Dietary pattern	-	-	-	0.220
Non-vegetarian	26.8	40.0	16.7	
Vegetarian	73.2	60.0	83.3	
Alcohol consumption	-	-	-	0.516
Never	81.7	82.0	100.0	
Ever	18.3	18.0	0.0	

* Mean and standard deviation.

**Table 2.** Percent difference in PI among those in the prediabetic and diabetic versus normal MBG range, age adjusted and multivariable adjusted models.

Parameter	Age-adjusted	Multivariable adjusted
	Change in PI % (95% CI)	Change in PI % (95% CI)
Normal MBG range (<117 mg/dL)	0.0	0.0
Prediabetic MBG range (117–137 mg/dL)	−13.0 (−29.5, 3.4)	−12.9 (−30.5, 4.8)
Diabetic MBG range (>137 mg/DL)	−16.0 (−54.2, 22.0)	−20.4 (−59.2, 18.4)
*p* for trend	0.109	0.119
R^2^	0.053	0.131

**Table 3.** Percent difference in PI among covariates in multivariable adjusted model*.

Parameter	% Difference in PI	95% CI
Age at enrollment		
50–69	0.0	0.0
70–79	−8.1	−28.2, 11.9
80–89	−8.2	−31.0, 14.4
90+	−24.1	−63.9, 15.6
Gender		
Female	0.0	0.0
Male	9.3	−8.2, 2.7
Race		
Whites	6.8	−11.5, 25.2
Blacks	0.0	0.0
BMI		
<25	0.0	0.0
25–30	−6.2	−25.4, 12.9
>30	−9.0	−33.2, 15.2
Exercise		
≤1 hr/wk	0.0	0.0
>1 hr/wk	12.6	−5.7, 30.9
Dietary Pattern		
Vegetarian	−7.2	−26.1, 11.7
Non-vegetarian	0.0	0.0
Alcohol consumption		
Never	0.0	0.0
Ever	−19.3	−41.7, 3.1

* Adjusted for MBG, age, gender, race, BMI, exercise, BMI, dietary pattern and alcohol consumption.
